# Supplementary material for: Epitome: predicting epigenetic events in novel cell types with multi-cell deep ensemble learning
Source: Nucleic Acids Res. 2021 Aug 11;49(19):e110. doi: 10.1093/nar/gkab676 (PMC8565335; doi:10.1093/nar/gkab676)
Supplement: gkab676_Supplemental_Files [file gkab676_supplemental_files.zip › supplement.pdf]

1 Supplementary Figures

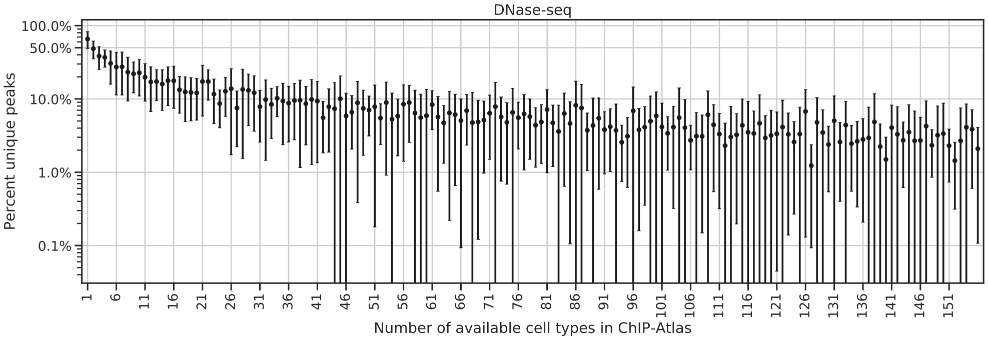

(a)

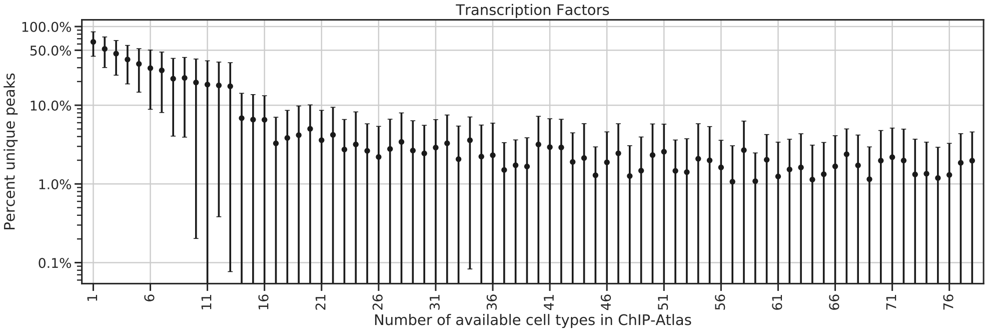

(b)

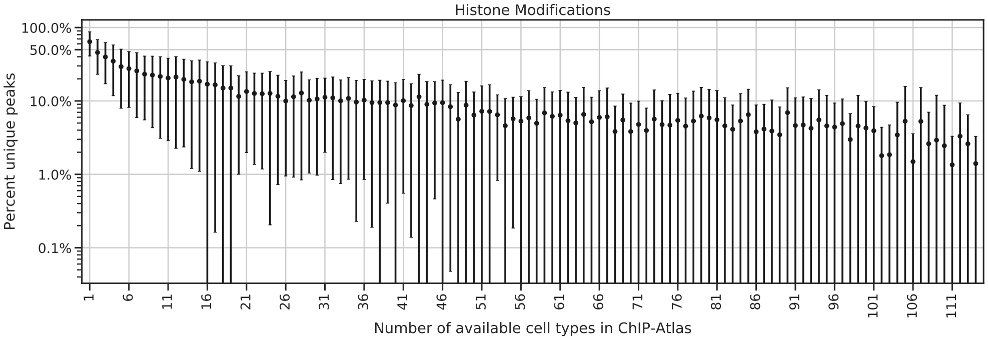

(c)

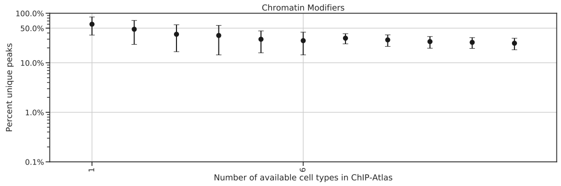

(d)

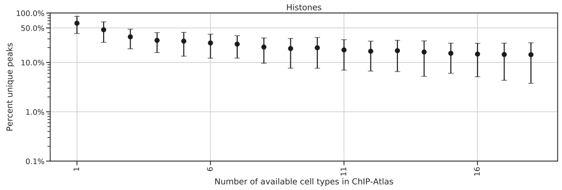

(e)

Figure S1: Related to Figure 1(a). Weighted means and standard deviations for percent of unique peaks observed in a cell type as the number of available cell types for a given epigenetic event increases. Means and standard deviations are weighted inversely proportional to the number of datapoints for a given target. Data curated from the ChIP-Atlas database [35]. Results are broken down into the following categories: (a) DNase-seq, (b) transcription factors, (c) histone modifications, (d) chromatin modifiers, and (e) histones.

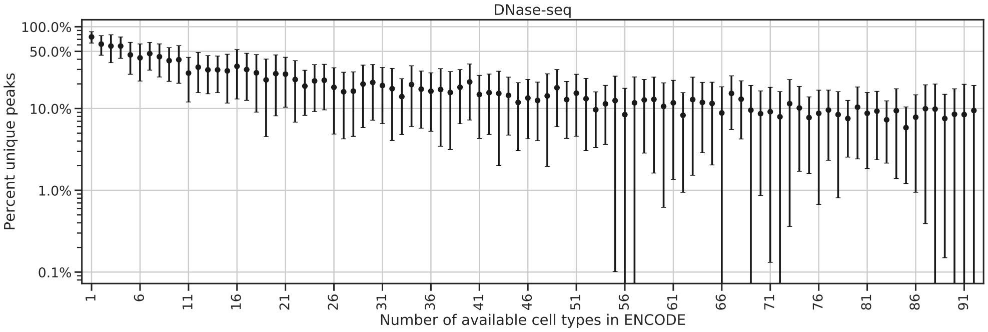

(a)

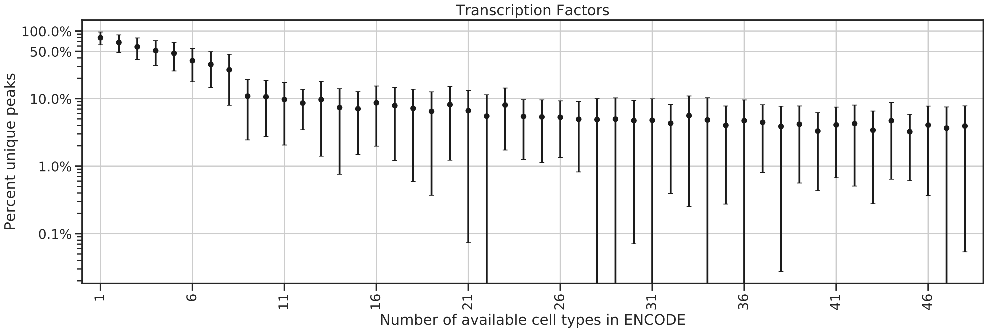

(b)

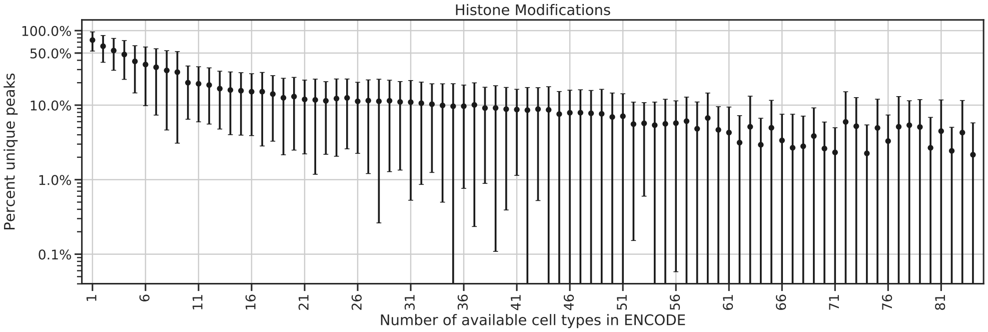

(c)

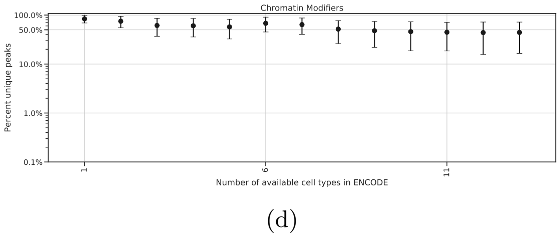

(d)

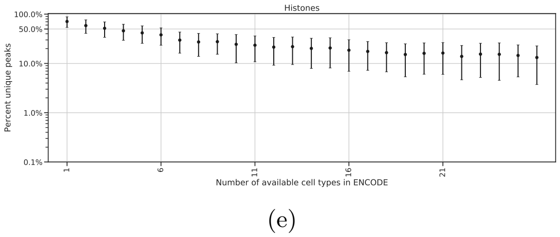

(e)

Figure S2: Related to Figure 1(a). Weighted means and standard deviations for percent of unique peaks observed in a cell type as the number of available cell types for a given target increases. Means and standard deviations are weighted inversely proportional to the number of datapoints for a given target. Data curated from ENCODE 3 [1]. Results are broken down into the following categories: (a) DNase-seq, (b) transcription factors, (c) histone modifications, (d) chromatin modifiers, and (e) histones.

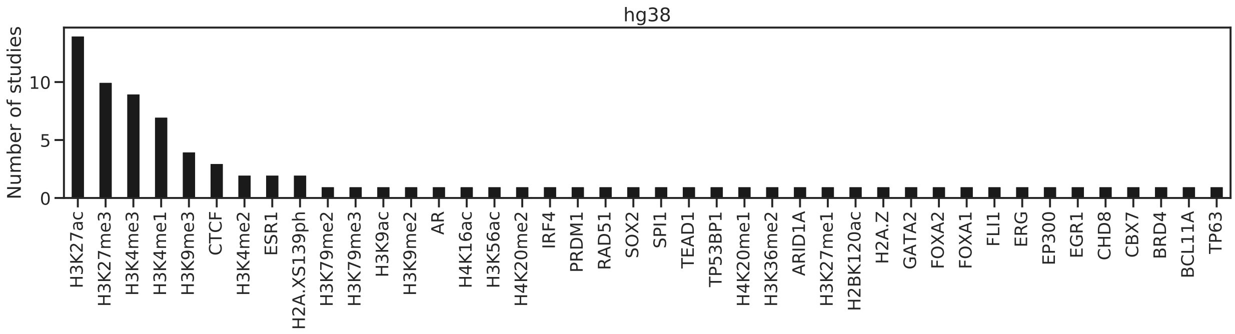

Figure S3: Frequency at which a given ChIP-seq target is present in a study included in ChIP-Atlas [35] that contains at least two ChIP-seq experiments. Only includes studies aligned and processed under the hg38 genome.

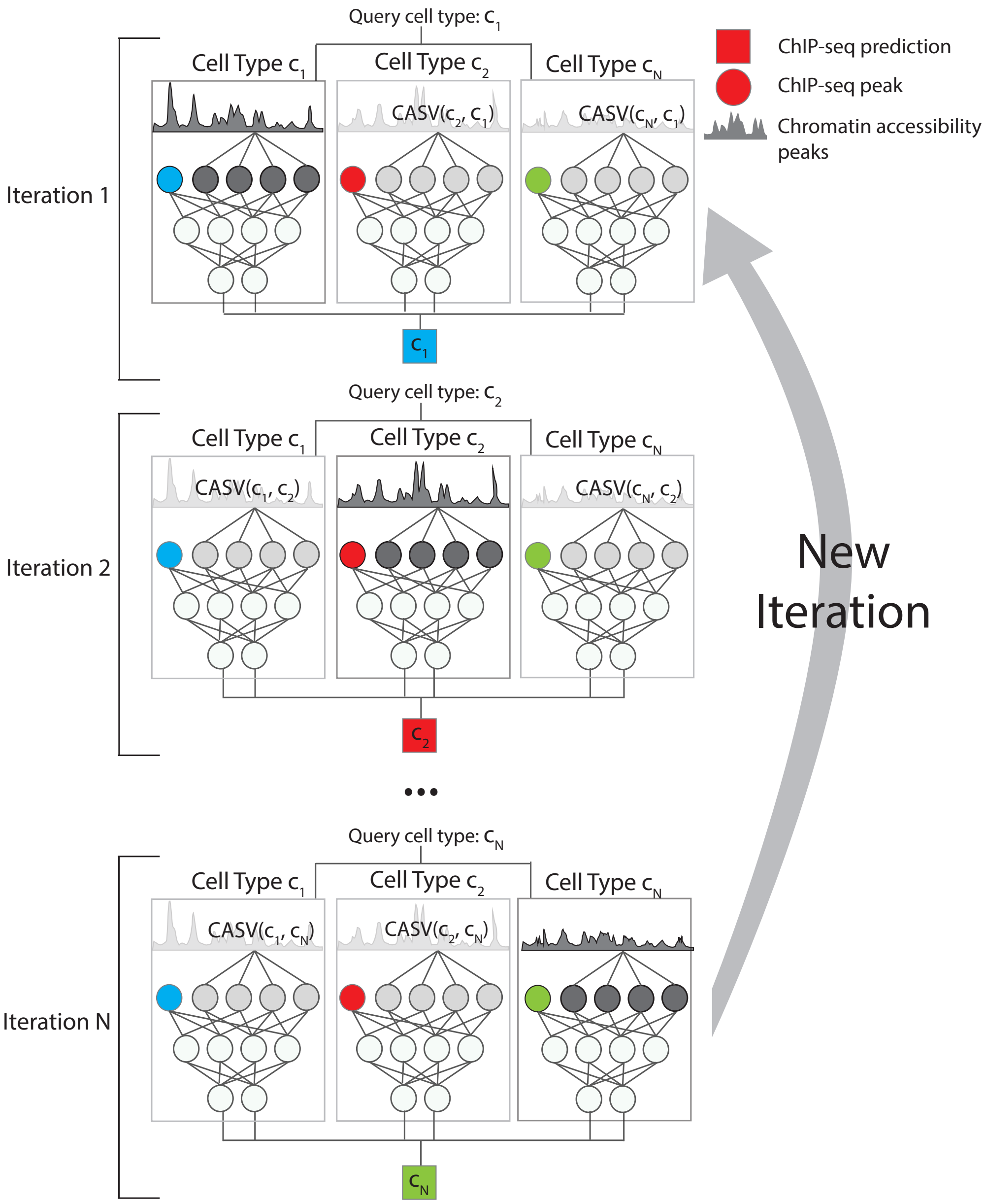

Figure S4: Related to Figure 1. Visual demonstration of cellular setting rotation mechanism used for training Epitome models. Epitome iteratively rotates through which ENCODE cellular setting is used as labels to predict ChIP-seq peaks in a given genomic loci. Remaining cellular settings are used as features. Once all cellular settings in a given genomic loci have been used as labels, a new genomic region is chosen, and all cellular settings are again rotated through to be used as labels.

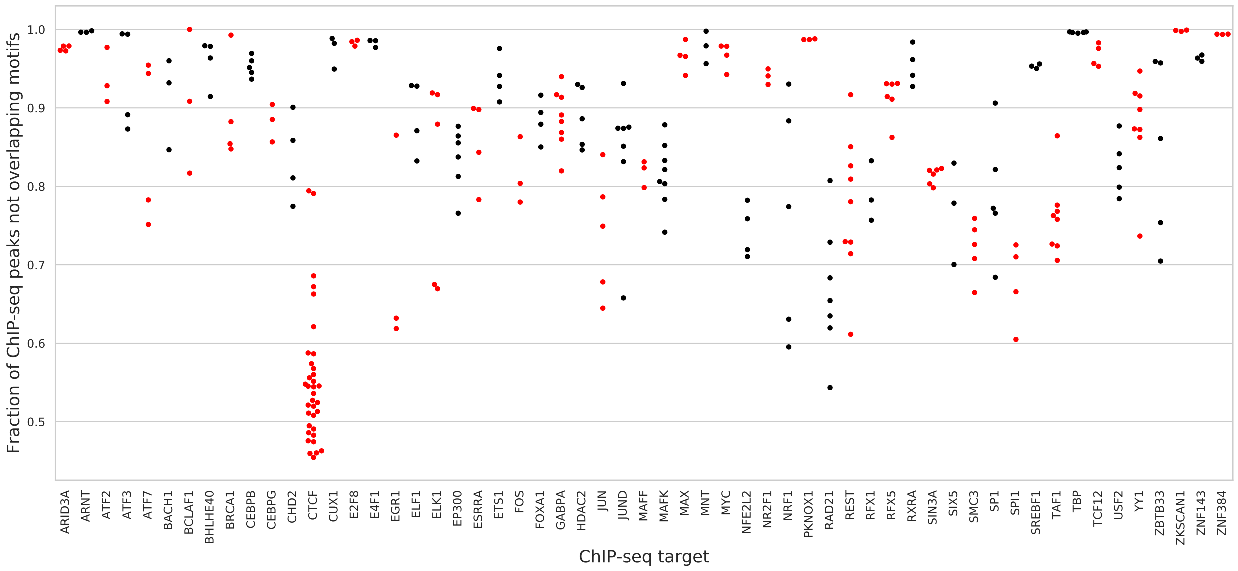

Figure S5: Related to Figure 2. Ratios of ChIP-seq peaks from ENCODE (hg38) that do not overlap any motif. 77 TFs and chromatin modifiers were considered across 40 cell types (Supplementary Table S6). Each data point represents the ratio of motif misses for a ChIP-seq experiment from a TF/chromatin modifier and cell type combination.

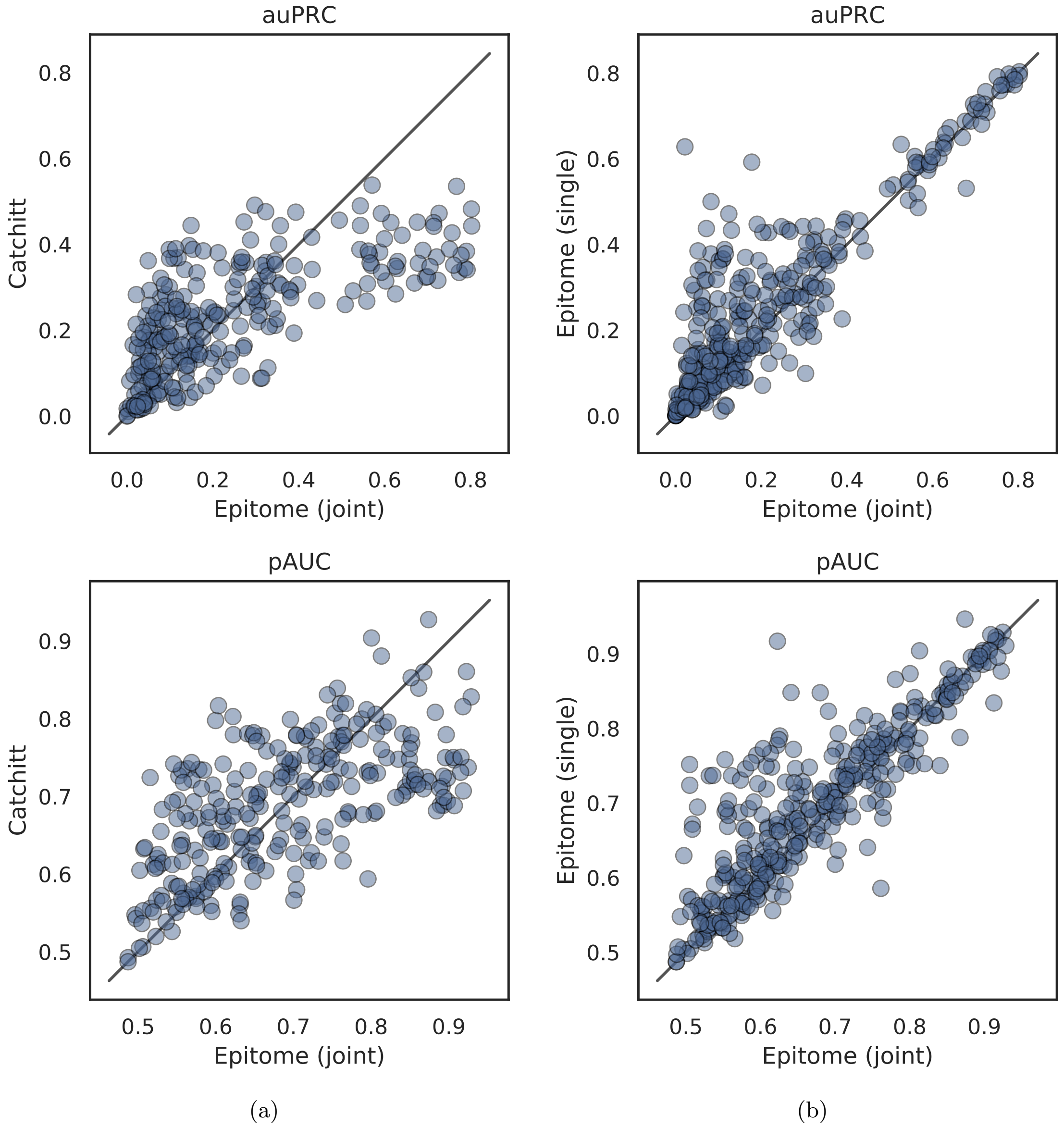

Figure S6: Related to Figure 2. Performance of Epitome joint models, single models, and Catchitt for predicting ChIP-seq peaks for 77 transcription factors on chromosomes 8 and 9 in 40 held out primary cells, tissues, and cell lines. (a) auPRC and pAUC (5% FPR) scores for Epitome joint models and Catchitt. (b) auPRC and pAUC (5% FPR) scores for Epitome models trained jointly and Epitome models trained individually (single) for each TF.

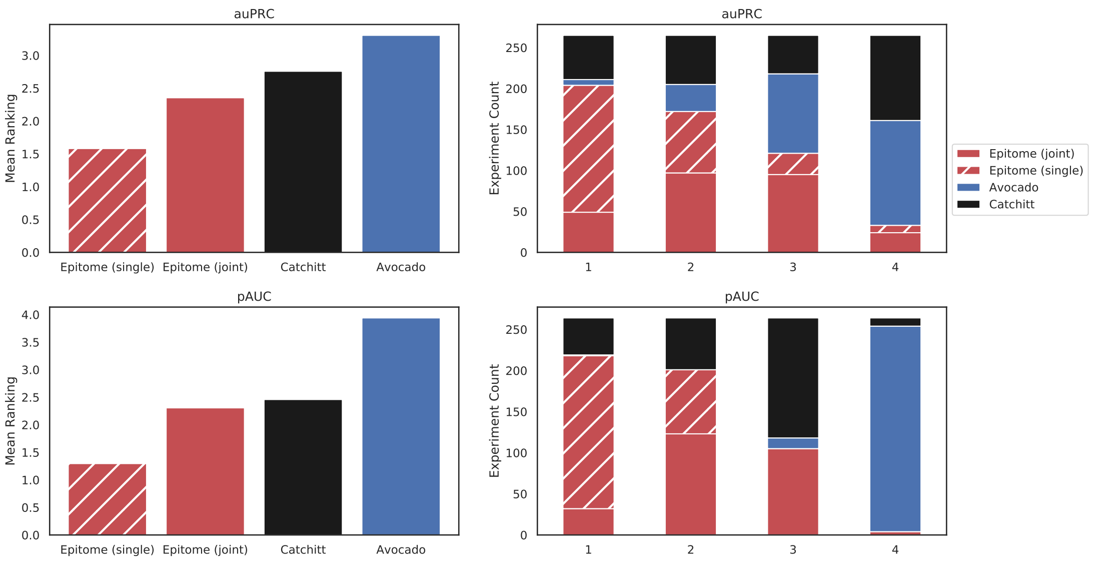

(a)

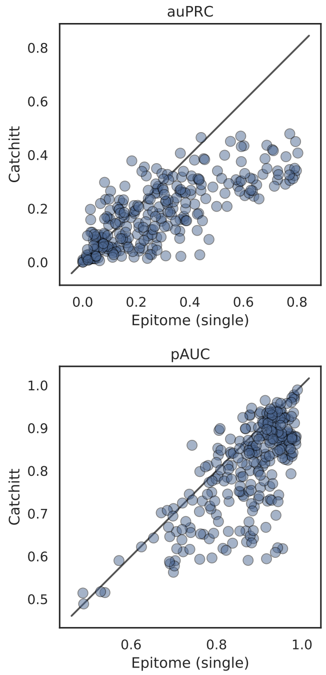

(b)

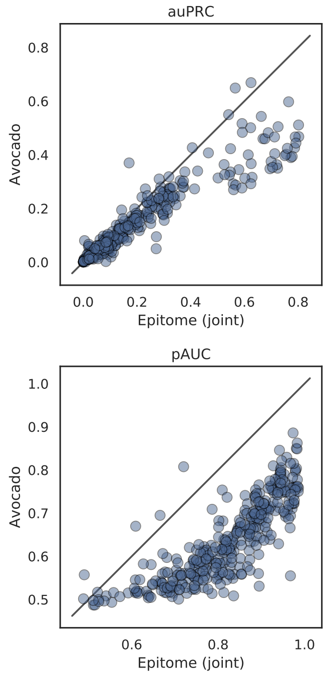

(c)

Figure S7: Related to Figure 2. Comparison of Epitome, Avocado, and Catchitt for predicting transcription factor binding sites (TFBS) for 77 transcription factors (TFs) in 40 primary cells, cell lines, and tissues from ENCODE evaluated across all 200bp regions on chromosomes 8 and 9. (a) Frequency at which each method obtains a rank for predicting TFBS across 77 transcription factors in 40 held out cell lines, tissues, and primary cells, totaling 264 comparisons. Evaluated methods include Avocado [30], Catchitt [24], a joint Epitome model, and single Epitome models, where each TF is trained separately. (Left) Mean pAUC (5% FPR) and auPRC ranking for each method. (Right) Frequency at which each method obtains a rank based on pAUC and auPRC. (b) Scatter plots comparing auPRC and pAUC (5% FPR) between Epitome and Catchitt. Both Catchitt and Epitome trained individual models for each TF evaluated. (c) Scatter plots comparing auPRC and pAUC (5% FPR) between Epitome and Avocado. Both Avocado and Epitome trained joint models for all TFs evaluated.

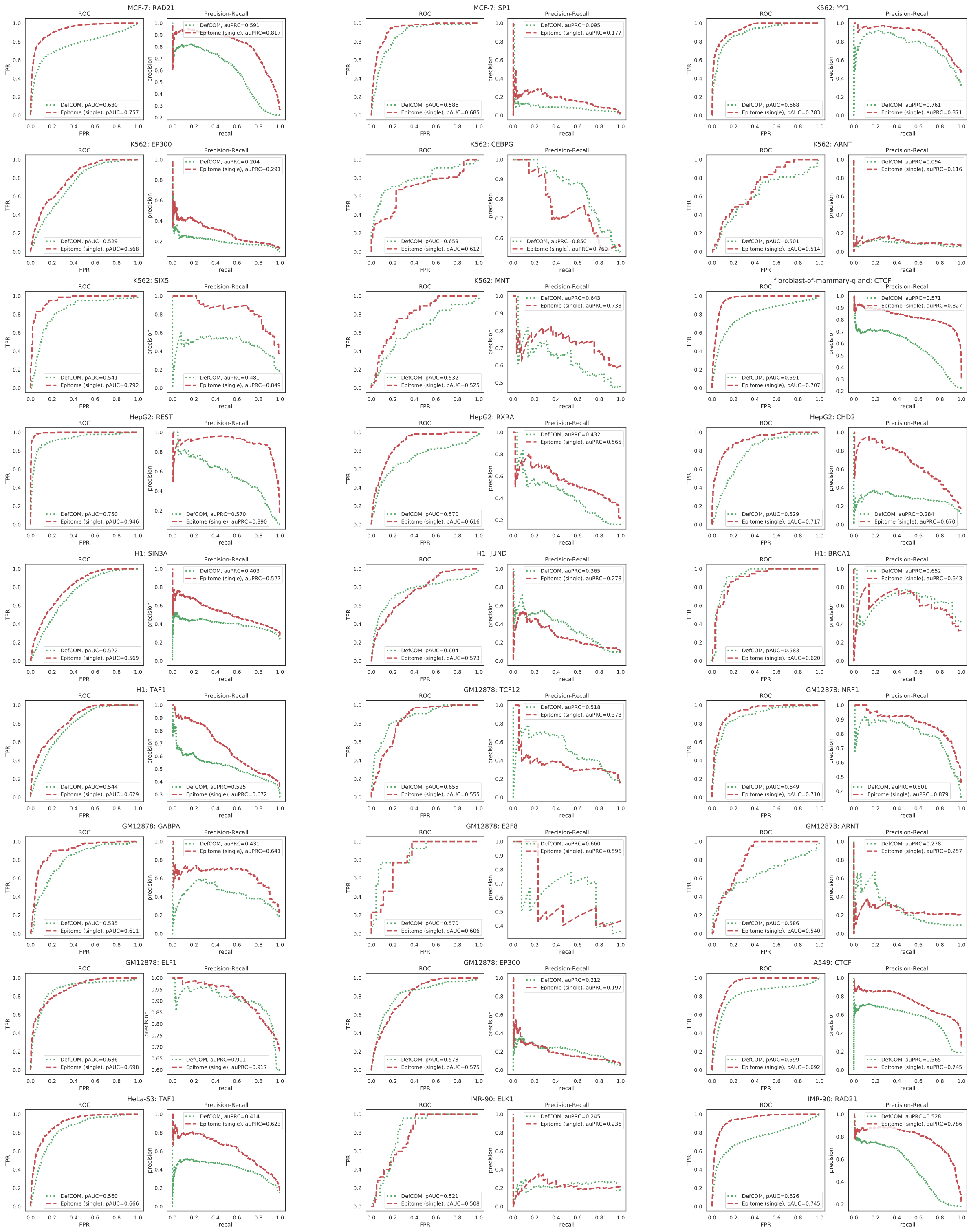

Figure S8: Related to Figure 2. Example ROC and PR curves for Epitome single models and DefCOM. Each plot represents evaluation of a randomly selected transcription factor in a held out cell line, primary cell, or tissue. All models were trained and evaluated on ENCODE processed peaks from the hg38 genome and were evaluated on regions that overlap a motif specific to the TF being evaluated.

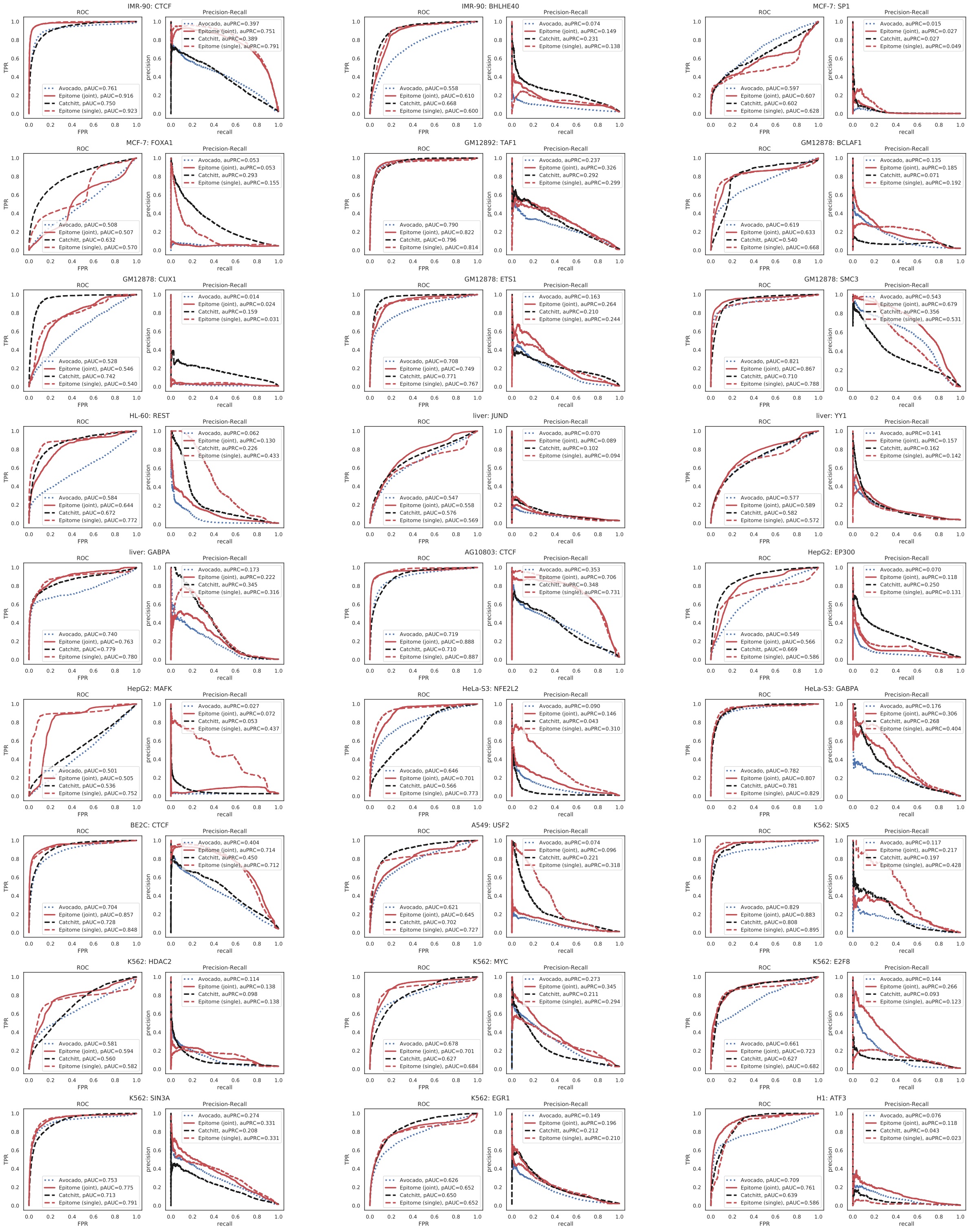

Figure S9: Related to Figure 2. Example ROC and PR curves for Avocado, Catchitt, Epitome single models, and Epitome joint models. Each plot represents evaluation of a randomly selected transcription factor in a held out cell line, primary cell, or tissue. All models were trained and evaluated on ENCODE processed peaks from the hg38 genome and were evaluated on chromosomes 8 and 9.

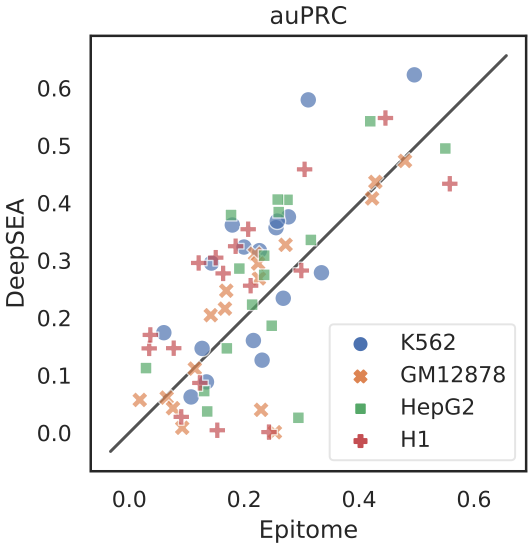

(a)

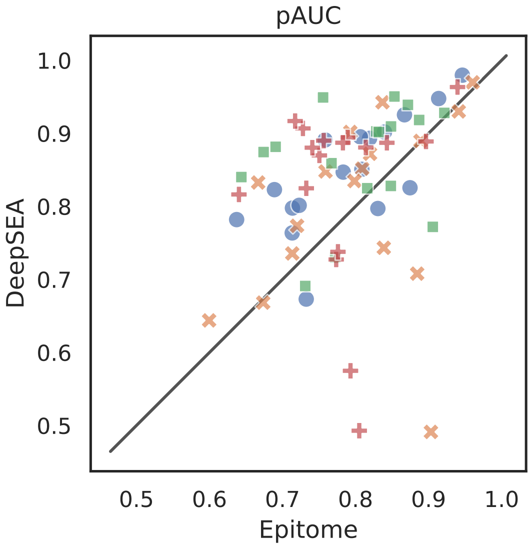

(b)

Figure S10: Related to Figure 2. Performance metrics of Epitome and DeepSEA for predicting ChIP-seq peaks for 17 transcription factors on chromosomes 8 and 9 in four held out cell lines, resulting in 68 comparisons. Four held out cell lines include K562, GM12878, HepG2, and H1. Transcription factors compared include: CEBPB, CHD2, CTCF, EP300, GABPA, JUND, MAFK, MAX, MYC, NRF1, RAD21, REST, RFX5, SRF, TAF1, TBP, and USF2. (a) auPRC and (b) pAUC (5% FPR) scores for Epitome and DeepSEA.

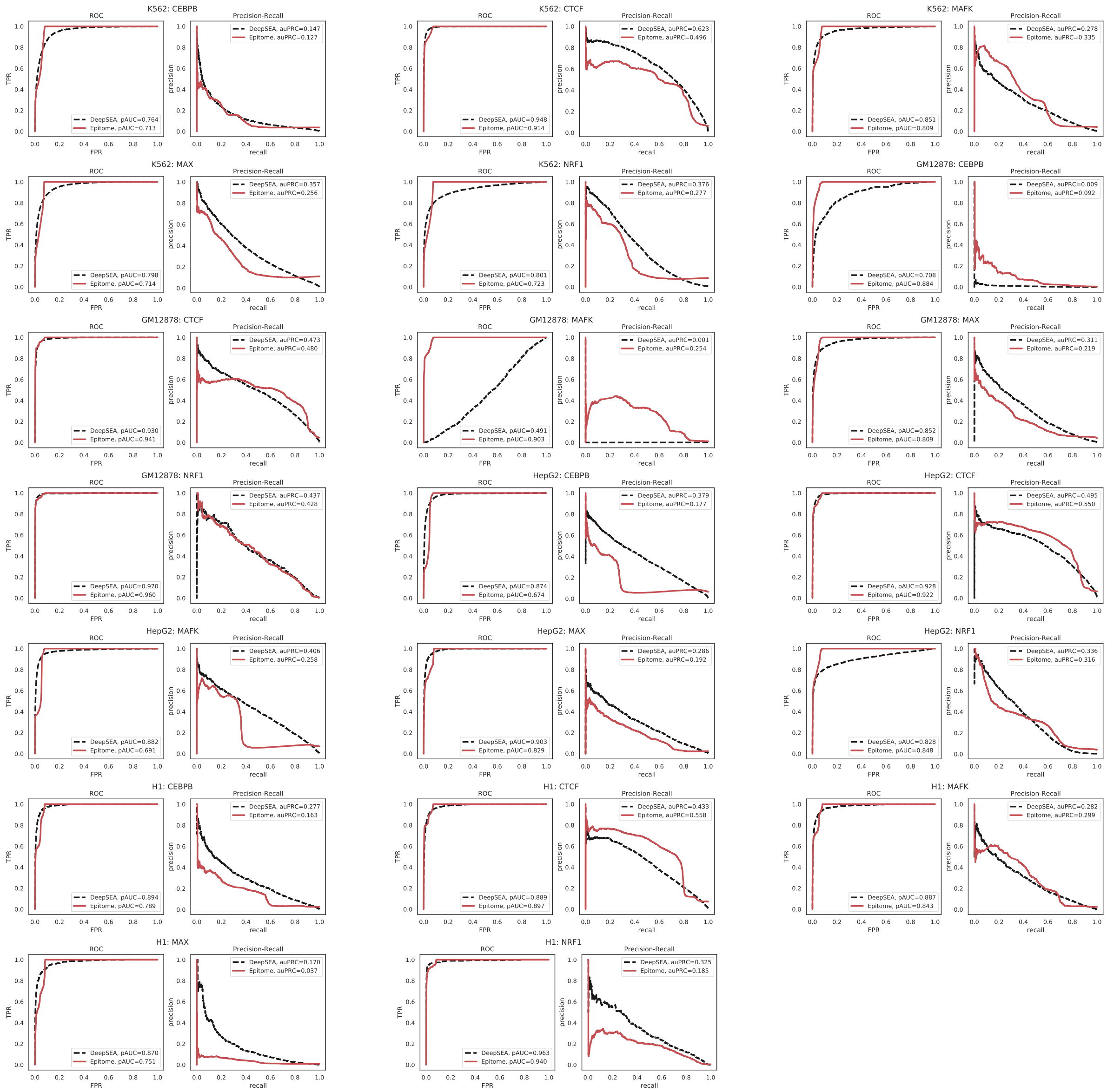

Figure S11: Related to Figure S10. Example ROC and PR curves for DeepSEA and Epitome joint models. Each plot represents evaluation of a randomly selected transcription factor in a held out cell line. All models were trained and evaluated on ENCODE processed peaks from the hg19 genome and were evaluated on chromosomes 8 and 9.

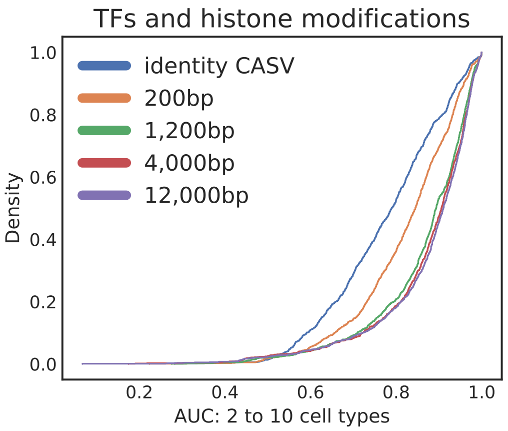

(a)

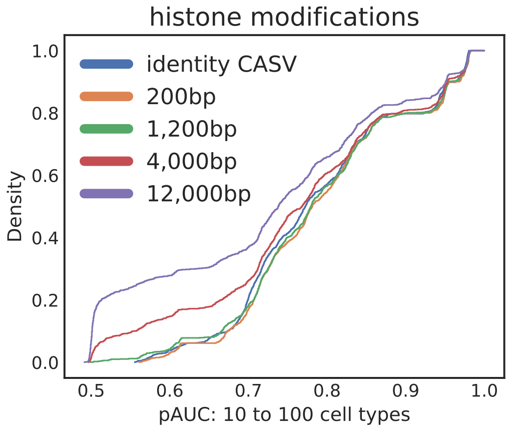

(b)

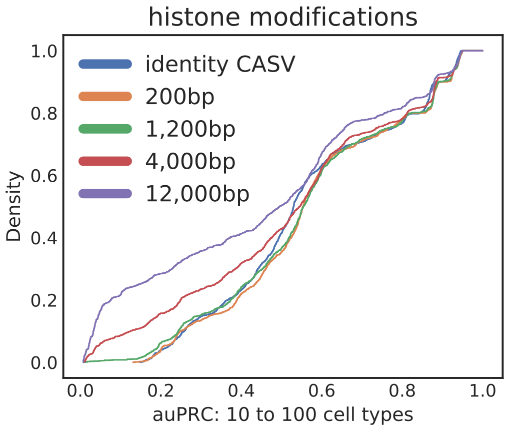

(c)

Figure S12: Related to Figure 4. Considering genomic contexts of various sizes to compute cell type similarity in the CASV affects performance of transcription factors (TFs) and histone modifications. Various DNase-seq window sizes are considered for computing the chromatin accessibility vector (CASV). Only DNase-seq is used to compute cell type similarity in the CASV. DNase-seq window sizes considered include no DNase-seq, 200bp, 1,200bp, 4,000bp, and 12,000bp around a peak of interest. (a) Cumulative distribution functions (CDFs) of Epitome performance in terms of area under the receiver operating characteristic curve (AUC) for TFs and histone modifications in Epitome models trained on 2 to 10 cell types.(b),(c) CDFs of Epitome performance for histone modifications in Epitome models trained on more than 10 cell types. Performance was measured in (b) partial area under the receiver operating characteristic curve (pAUC) (5% FPR) and (c) area under the precision recall curve (auPRC).

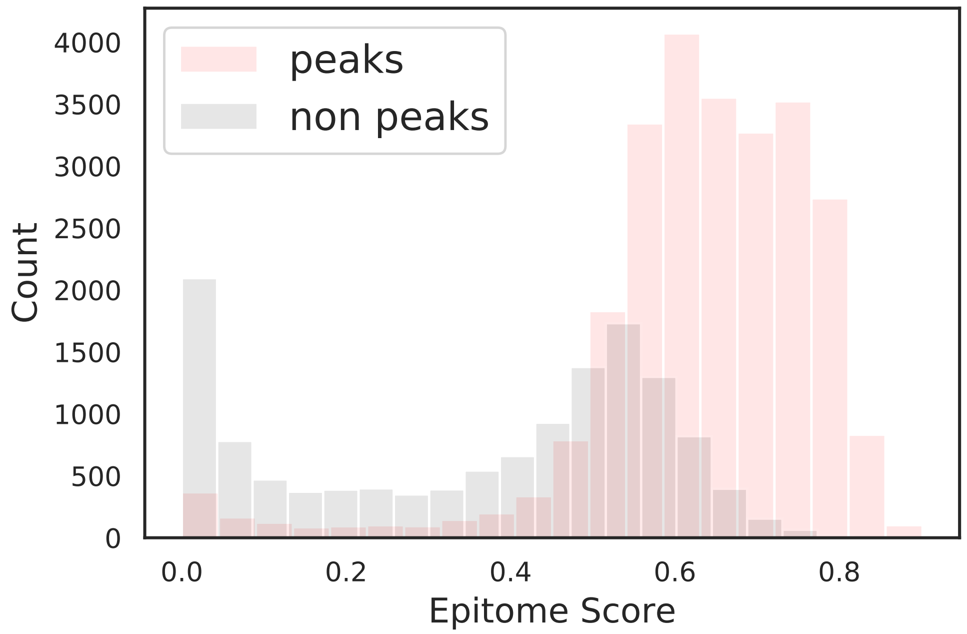

(a)

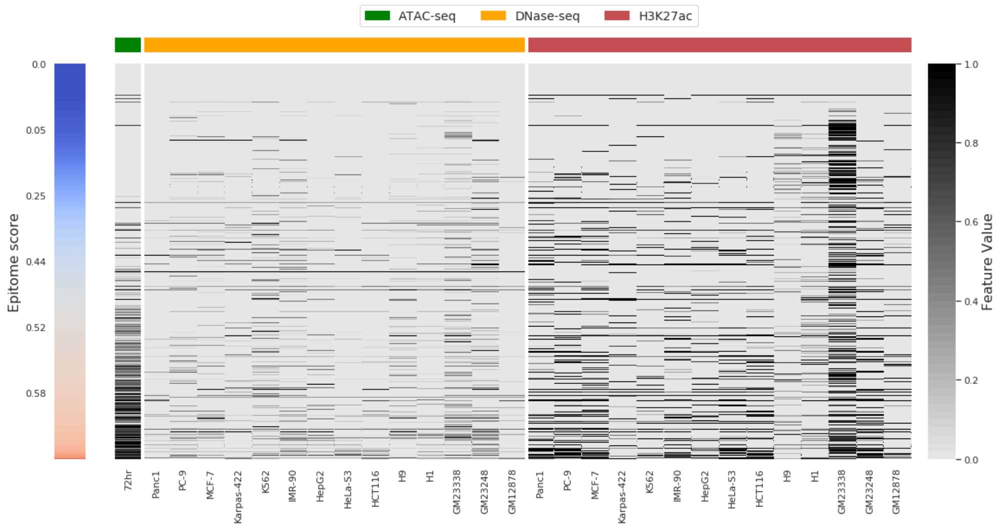

(b)

Figure S13: Related to Figure 5. (a) Epitome predictions of H3K27ac peaks at 72hr after neural induction for peak and nonpeak regions. (b) Heatmap of features used by Epitome for 13,248 regions that do not contain H3K27ac peaks at 72hr. Color bar on left represents Epitome scores, where blue represents true negatives and red represents false positives.

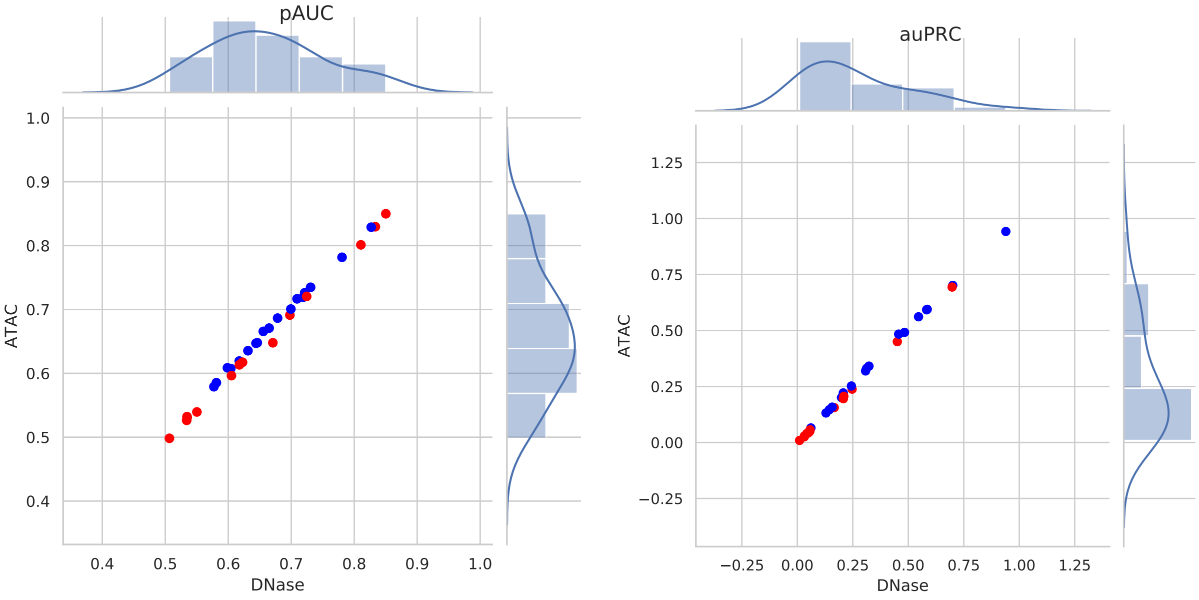

(a)

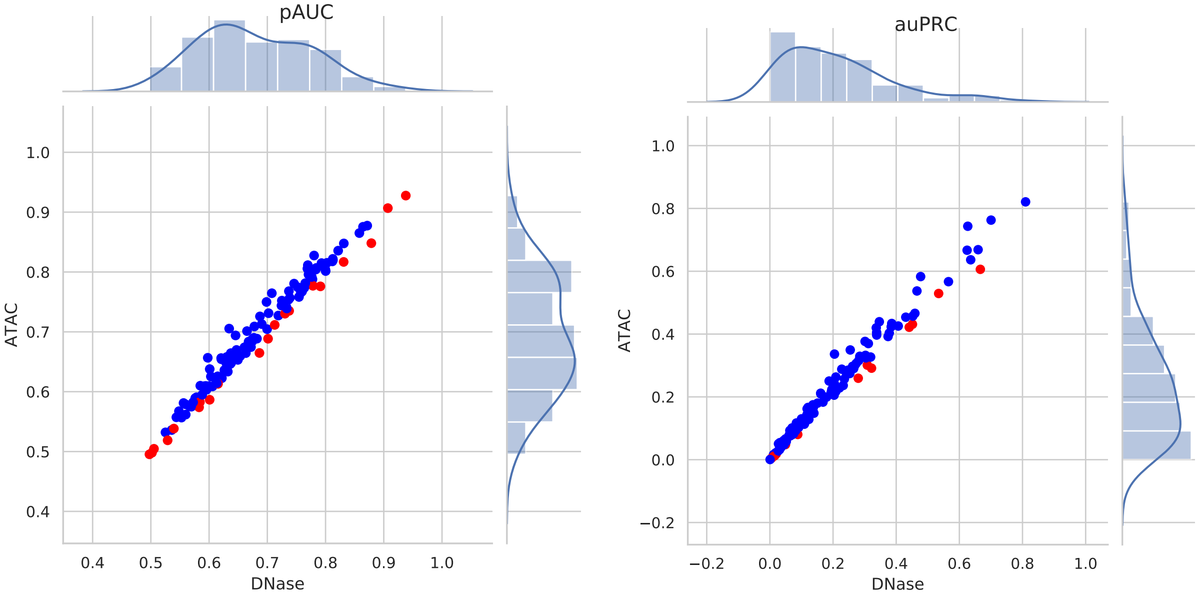

(b)

Figure S14: Epitome performs comparably when training models using DNase-seq and evaluating on a new cell line using ATAC-seq. (a) Comparative pAUC (5% FPR) and auPRC performance of 33 TFs when predicting genome wide binding in the A549 cell line using an Epitome model trained using DNase-seq. x axis shows pAUC (left) and auPRC (right) using ENCODE A549 DNase-seq during evaluation, and y axis shows pAUC (left) and auPRC (right) using ENCODE ATAC-seq during evaluation. Blue indicates TFs that perform better when predicted using ATAC-seq data during evaluation. Red indicates TFs that perform better when predicted using DNase-seq data during evaluation. (b) Comparative pAUC (5% FPR) and auPRC performance of 128 TFs when predicting genome wide binding in the K562 cell line using an Epitome model trained using DNase-seq. x axis shows pAUC (left) and auPRC (right) using ENCODE K562 DNase-seq during evaluation, and y axis shows pAUC (left) and auPRC (right) using ENCODE ATAC-seq during evaluation.

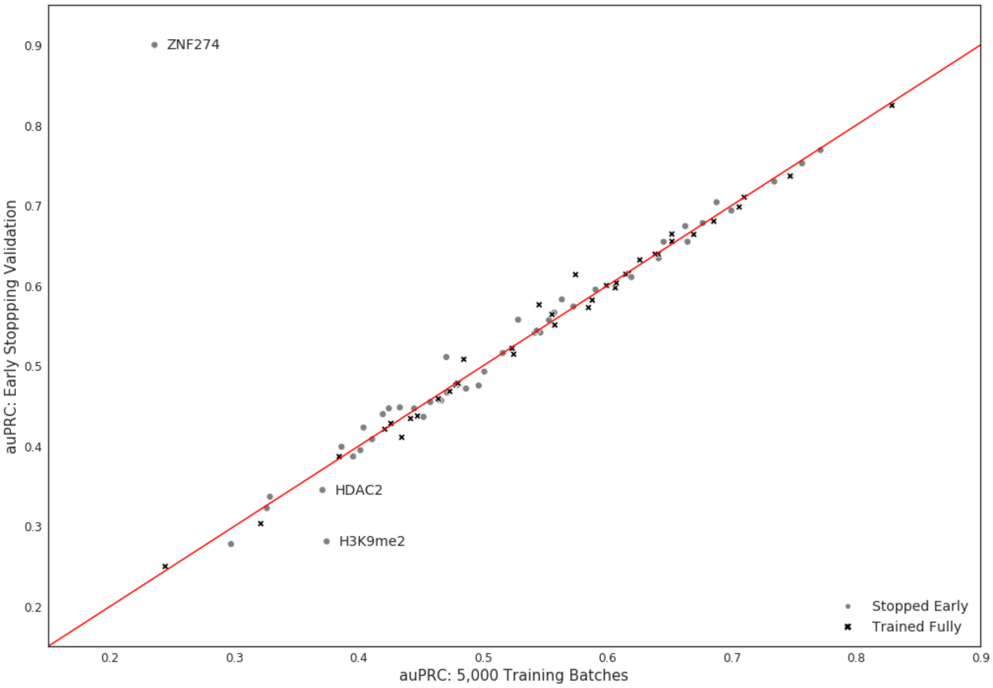

(a)

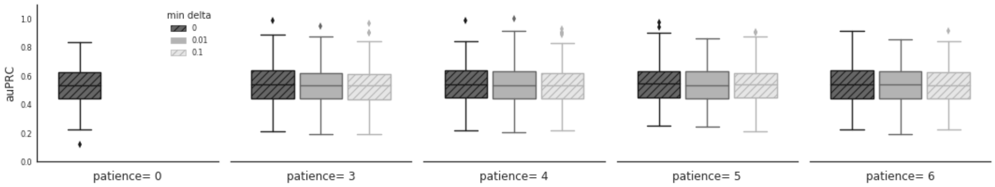

(b)

Figure S15: Performance of single TF Epitome models with and without early stopping validation. Values are area under the precision recall curve (auPRC). Models trained without the early stopping validation method trained for 5,000 batches. 5 models were trained for each combination of parameters using early stopping validation method while 5 models were trained without early stop validation. (a) Median auPRC performance of single TF Epitome models with and without early stopping validation, using hyper-parameters of a patience of 5 and minimum delta of 0. The median auPRC is computed across 5 models. 44 out of the 85 TFs stopped training early (before 5,000 batches), with an overall mean of 4,300 training batches. (b) auPRC of single TF Epitome models on different minimum delta and patience hyper-parameters. Each sub-plot indicates a different patience hyper-parameter (the x-axis), and each bar plot hue indicates a different min delta hyper-parameter. The first sub-plot with a 0 patience and 0 min delta is the performance from the baseline model, which was trained on 5,000 training batches (without stopping early).

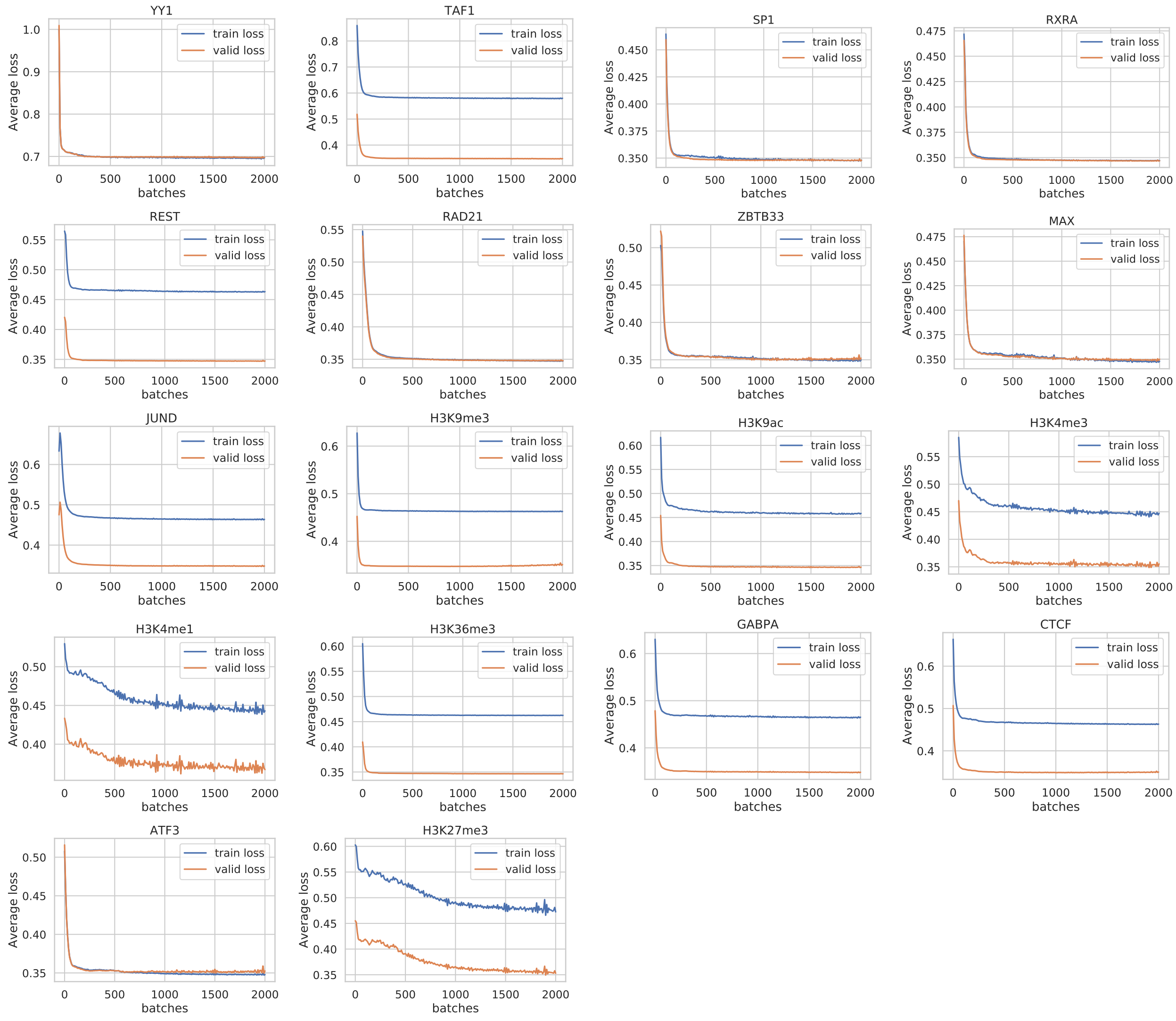

Figure S16: Average training and validation loss for 18 ChIP-seq targets, including transcription factors, chromatin modifiers and histone modifications. All ChIP-seq targets were trained jointly. Average train loss was calculated from 10,000 sampled points from the training dataset. Valid loss was calculated from all points on chromosome 7 meeting the sampling criteria as described in Section 4.3.4. Average loss is calculated as the sigmoid cross entropy, averaged across all evaluated data points (See Equation 2). The model was trained for 2000 iterations, without early stopping.

2 Supplementary Tables and Captions

Name	Comparison included in manuscript	Method	Features Used	Predicts
**Epitome**	yes	Neural Network	DNase-seq and<br>ChIP-seq (binary peaks)	TFBS<br>histone modifications
DeepSEA [19]	yes	Convolutional Neural Network	DNA sequence	TFBS<br>histone modifications<br>DNase I–hypersensitive sites
BindSpace [20]	no	StarSpace [64]	DNA sequence	TFBS
Catchitt [24]	yes	Product of independent densities and discrete distributions	motifs and DNase-seq<br>(fold enrichment coverage and peaks)	TFBS
DefCOM [23]	yes	Support Vector Machine (SVM)	motifs and DNase-seq<br>(DNase1 digestion sites)	TFBS
Anchor [51]	no	XGBoost [65]	motifs and DNase-seq<br>(fold enrichment coverage and read alignments)	TFBS
Avocado [30]	yes	Deep tensor factorization	genomic regions,<br>DNase-seq, ChIP-seq<br>(fold change over control signal p-value)	TFBS<br>histone modifications<br>DNase I–hypersensitive sites

Table S1: List of methods for predicting transcription factor binding sites (TFBS). Related to Figure 2. Methods compared include Epitome, DeepSEA, Catchitt, DefCOM, and Avocado.

Table S2: List of ChIP-seq and DNase-seq ENCODE accession numbers used for training and validation of models.

Table S3: List of ChIP-seq and DNase-seq ChIP-Atlas accession numbers used for validation of models and calculating the fraction of unique peaks in Figure 1(a).

Table S4: Accession numbers for ATAC-seq used for validation of models and comparison to DNase-seq trained models. Related to Supplementary Figure S14.

Table S5: Motifs used for training and validating DefCoM [23] and Catchitt [24]. Motifs were taken from Cis-BP [55] and Kheradpour et al. [56].

Table S6: Cell types and ChIP-seq targets used for evaluation of Epitome, Avocado, Catchitt, and DefCoM. Related to Figure 2.

Table S7: Area under the precision recall (auPRC) and partial area under the receiver operating characteristic curve (5% FPR threshold, auROC) for DefCoM and Epitome in regions overlapping motifs.

Table S8: Area under the precision recall (auPRC) and partial area under the receiver operating characteristic curve (5% FPR threshold, auROC) for Epitome, Avocado, and Catchitt. Related to Figure 2.

Table S9:Area under the precision recall (auPRC) and partial area under the receiver operating characteristic curve (5% FPR threshold, auROC) for Epitome and DeepSEA. Related to Figure S10.

Table S10: Number of ChIP-seq peaks that overlap motifs for 77 ChIP-seq targets. Related to Supplementary Figure S5.

References

[1] Federico Abascal et al. "Expanded encyclopaedias of DNA elements in the human and mouse genomes". In: *Nature* 583.7818 (2020), pp. 699–710.

[2] ENCODE Project Consortium et al. "The ENCODE (ENCyclopedia of DNA elements) project". In: *Science* 306.5696 (2004), pp. 636–640.

[3] Anshul Kundaje et al. "Integrative analysis of 111 reference human epigenomes". In: *Nature* 518.7539 (2015), pp. 317–330.

[4] Albert J Keung et al. "Using targeted chromatin regulators to engineer combinatorial and spatial transcriptional regulation." In: *Cell* 158.1 (2014), pp. 110–120.

[5] Bing Li, Michael Carey, and Jerry L Workman. "The role of chromatin during transcription." In: *Cell* 128.4 (2007), pp. 707–719.

[6] Shelley L. Berger. "The complex language of chromatin regulation during transcription". In: *Nature* 447.7143 (2007), pp. 407–412.

[7] Thomas Jenuwein and C. David Allis. "Translating the Histone Code". In: *Science* 293.5532 (2001), pp. 1074–1080.

[8] D. Wang et al. "Transcription factor co-localization patterns affect human cell type-specific gene expression". In: *BMC Genomics* 13 (2012), p. 263.

[9] Y Zhang and D Reinberg. "Transcription regulation by histone methylation: interplay between different covalent modifications of the core histone tails." eng. In: *Genes Dev* 15.18 (2001), pp. 2343–2360.

[10] Alan P Boyle et al. "High-resolution mapping and characterization of open chromatin across the genome". In: *Cell* 132.2 (Jan. 2008), pp. 311–322.

[11] Jason D Buenrostro et al. "Transposition of native chromatin for fast and sensitive epigenomic profiling of open chromatin, DNA-binding proteins and nucleosome position". In: *Nature methods* 10.12 (2013), pp. 1213–1218.

[12] Robin Andersson and Albin Sandelin. "Determinants of enhancer and promoter activities of regulatory elements". In: *Nature Reviews Genetics* 21.2 (2020), pp. 71–87.

[13] Ailing Chen, Daozhen Chen, and Ying Chen. "Advances of DNase-seq for mapping active gene regulatory elements across the genome in animals". In: *Gene* 667 (2018), pp. 83–94.

[14] Robert E. Thurman et al. "The accessible chromatin landscape of the human genome". In: *Nature* 489.7414 (2012), pp. 75–82.

[15] Debasish Raha, Miyoung Hong, and Michael Snyder. "ChIP-Seq: A method for global identification of regulatory elements in the genome". In: *Current protocols in molecular biology* 91.1 (2010), pp. 21–19.

[16] Peter J Skene and Steven Henikoff. "An efficient targeted nuclease strategy for high-resolution mapping of DNA binding sites". In: *eLife* 6 (2017), e21856.

[17] Jeff Vierstra and John A Stamatoyannopoulos. "Genomic footprinting". In: *Nature Methods* 13.3 (2016), pp. 213–221.

[18] Babak Alipanahi et al. "Predicting the sequence specificities of DNA- and RNA-binding proteins by deep learning." In: *Nature biotechnology* 33 (8 2015), pp. 831–838.

[19] Jian Zhou and Olga G Troyanskaya. "Predicting effects of noncoding variants with deep learning-based sequence model". In: *Nature methods* 12.10 (2015), pp. 931–934.

[20] Han Yuan et al. "BindSpace decodes transcription factor binding signals by large-scale sequence embedding". In: *Nature Methods* 16.9 (2019), pp. 858–861.

[21] M. Setty and CS Leslie. "SeqGL identifies context-dependent binding signals in genome-wide regulatory element maps". In: *PLoS Computational Biology* 11.5 (2015), e1004271.

[22] Matan Goldshtein et al. "Transcription Factor Binding in Embryonic Stem Cells Is Constrained by DNA Sequence Repeat Symmetry". In: *Biophysical Journal* 118.8 (2020), pp. 2015–2026.

[23] Bryan Quach and Terrence S Furey. “DeFCoM: analysis and modeling of transcription factor binding sites using a motif-centric genomic footprinter”. In: *Bioinformatics* 33.7 (2016), pp. 956–963.

[24] Jens Keilwagen, Stefan Posch, and Jan Grau. “Accurate prediction of cell type-specific transcription factor binding”. In: *Genome Biology* 20.1 (2019), p. 9.

[25] Juhani Kähärä and Harri Lähdesmäki. “BinDNase: a discriminatory approach for transcription factor binding prediction using DNase I hypersensitivity data”. In: *Bioinformatics* 31.17 (2015), pp. 2852–2859.

[26] Anil Raj et al. “msCentipede: Modeling Heterogeneity across Genomic Sites and Replicates Improves Accuracy in the Inference of Transcription Factor Binding”. In: *PloS one* 10.9 (2015), e0138030–e0138030.

[27] Wouter Meuleman et al. “Index and biological spectrum of human DNase I hypersensitive sites”. In: *Nature* 584.7820 (2020), pp. 244–251.

[28] Shane Neph et al. “An expansive human regulatory lexicon encoded in transcription factor footprints”. In: *Nature* 489.7414 (2012), pp. 83–90.

[29] I. Dunhan, A. Kundaje, and S. et al. Aldred. “An integrated encyclopedia of DNA elements in the human genome.” In: *Nature* 489 (2012), pp. 57–74.

[30] Jacob Schreiber et al. “Avocado: a multi-scale deep tensor factorization method learns a latent representation of the human epigenome”. In: *Genome Biology* 21.1 (2020), p. 81.

[31] Jason Ernst and Manolis Kellis. “Large-scale imputation of epigenomic datasets for systematic annotation of diverse human tissues”. In: *Nature Biotechnology* 33.4 (2015), pp. 364–376.

[32] Timothy J. Durham et al. “PREDICTD PaRallel Epigenomics Data Imputation with Cloud-based Tensor Decomposition”. In: *Nature Communications* 9.1 (2018), p. 1402.

[33] Trevor Standley et al. “Which Tasks Should Be Learned Together in Multi-task Learning?” In: *Proceedings of the 37th International Conference on Machine Learning.* Ed. by Hal Daumé III and Aarti Singh. Vol. 119. Proceedings of Machine Learning Research. PMLR, 2020, pp. 9120–9132.

[34] Jeff Vierstra et al. “Global reference mapping of human transcription factor footprints”. In: *Nature* 583.7818 (2020), pp. 729–736.

[35] Shinya Oki et al. “ChIP-Atlas: a data-mining suite powered by full integration of public ChIP-seq data”. In: *EMBO reports* 19.12 (2018), e46255.

[36] A. Kundaje et al. “ENCODE-DREAM in vivo Transcription Factor Binding Site Prediction Challenge.” In: *Synapse* (2017).

[37] Cory Y McLean et al. “GREAT improves functional interpretation of cis-regulatory regions”. In: *Nature Biotechnology* 28.5 (2010), pp. 495–501.

[38] Hua Ma et al. “On use of partial area under the ROC curve for evaluation of diagnostic performance”. In: *Statistics in medicine* 32.20 (2013), pp. 3449–3458.

[39] Wenjie Shu et al. “Genome-wide analysis of the relationships between DNaseI HS, histone modifications and gene expression reveals distinct modes of chromatin domains”. In: *Nucleic acids research* 39.17 (2011), pp. 7428–7443.

[40] Sergiu Hart. *Shapley Value. In: Game Theory.* Reading, Massachusetts: Palgrave Macmillan, London, 1989, pp. 210–216.

[41] Fumitaka Inoue et al. “Identification and Massively Parallel Characterization of Regulatory Elements Driving Neural Induction”. In: *Cell Stem Cell* 5 (2019), pp. 713–727.

[42] Qijin Yin et al. “DeepHistone: a deep learning approach to predicting histone modifications.” In: *BMC Genomics* 20.Suppl 2 (2019), p. 193.

[43] Federico Abascal et al. “Perspectives on ENCODE”. In: *Nature* 583.7818 (2020), pp. 693–698.

[44] Nathan Boley. “idr”. In: *GitHub repository* (2017).

[45] Yong Zhang et al. “Model-based Analysis of ChIP-Seq (MACS)”. In: *Genome Biology* 9.9 (2008), R137.

[46] R. A. Quinlan and M. I. Hall. "BEDTools: a flexible suite of utilities for comparing genomic features". In: *Bioinformatics* 26.6 (2010), pp. 841–842.

[47] Yuichi Kodama, Martin Shumway, and Rasko Leinonen. "The Sequence Read Archive: Explosive growth of sequencing data". In: *Nucleic acids research* 40 (2012), pp. D54–6.

[48] Jacob Schreiber et al. "A pitfall for machine learning methods aiming to predict across cell types". In: *bioRxiv* (2019).

[49] Francisco Charte et al. "MLSMOTE: Approaching imbalanced multilabel learning through synthetic instance generation". In: *Knowl. Based Syst.* 89 (2015), pp. 385–397.

[50] Lutz Prechelt. "Early Stopping - But When?" In: *Neural Networks: Tricks of the Trade: Second Edition*. Ed. by Grégoire Montavon, Geneviève B. Orr, and Klaus-Robert Müller. Berlin, Heidelberg: Springer Berlin Heidelberg, 2012, pp. 53–67. ISBN: 978-3-642-35289-8.

[51] Daniel Quang Hongyang Li and Yuanfang Guan. "Anchor: trans-cell type prediction of transcription factor binding sites". In: *Genome Res.* 29 (2019), pp. 281–292.

[52] A. Géron. *Hands-On Machine Learning with Scikit-Learn, Keras, and TensorFlow: Concepts, Tools, and Techniques to Build Intelligent Systems*. O'Reilly Media, 2019. ISBN: 9781492032595.

[53] F. Pedregosa et al. "Scikit-learn: Machine Learning in Python". In: *Journal of Machine Learning Research* 12 (2011), pp. 2825–2830.

[54] D K McClish. "Analyzing a portion of the ROC curve." In: *Med Decis Making* 9.3 (1989), pp. 190–195.

[55] Matthew T Weirauch et al. "Determination and inference of eukaryotic transcription factor sequence specificity." In: *Cell* 158.6 (2014), pp. 1431–1443.

[56] Pouya Kheradpour and Manolis Kellis. "Systematic discovery and characterization of regulatory motifs in ENCODE TF binding experiments." In: *Nucleic Acids Res* 42.5 (2014), pp. 2976–2987.

[57] Charles E. Grant, Timothy L. Bailey, and William Stafford Noble. "FIMO: scanning for occurrences of a given motif". In: *Bioinformatics* 27.7 (2011), pp. 1017–1018.

[58] Žiga Avsec et al. "The Kipoi repository accelerates community exchange and reuse of predictive models for genomics". In: *Nature Biotechnology* 37.6 (2019), pp. 592–600.

[59] Michael Waskom et al. *mwaskom/seaborn: v0.8.1 (September 2017)*. Version v0.8.1. Sept. 2017. DOI: 10.5281/zenodo.883859.

[60] A S Hinrichs et al. "The UCSC Genome Browser Database: update 2006." eng. In: *Nucleic Acids Res* 34.Database issue (2006), pp. D590–8.

[61] Endre Bakken Stovner and Pål Sætrom. "PyRanges: efficient comparison of genomic intervals in Python". In: *Bioinformatics* 36.3 (2019), pp. 918–919.

[62] Martın Abadi et al. *TensorFlow: Large-Scale Machine Learning on Heterogeneous Systems*. Software available from tensorflow.org. 2015.

[63] J. D. Hunter. "Matplotlib: A 2D graphics environment". In: *Computing in Science & Engineering* 9.3 (2007), pp. 90–95.

[64] Ledell Yu Wu et al. "StarSpace: Embed All The Things!" In: *AAAI*. 2018, pp. 5569–5577.

[65] Tianqi Chen and Carlos Guestrin. "XGBoost". In: *Proceedings of the 22nd ACM SIGKDD International Conference on Knowledge Discovery and Data Mining* (2016).
